# Valorization of Polyethylene Terephthalate to Muconic Acid by Engineering *Pseudomonas Putida*

**DOI:** 10.3390/ijms231910997

**Published:** 2022-09-20

**Authors:** Pan Liu, Yi Zheng, Yingbo Yuan, Tong Zhang, Qingbin Li, Quanfeng Liang, Tianyuan Su, Qingsheng Qi

**Affiliations:** State Key Laboratory of Microbial Technology, Shandong University, Qingdao 266237, China

**Keywords:** metabolic engineering, LCC, polyethylene terephthalate, enzymatic hydrolysis, bioconversion

## Abstract

Plastic waste is rapidly accumulating in the environment and becoming a huge global challenge. Many studies have highlighted the role of microbial metabolic engineering for the valorization of polyethylene terephthalate (PET) waste. In this study, we proposed a new conceptual scheme for upcycling of PET. We constructed a multifunctional *Pseudomonas putida* KT2440 to simultaneously secrete PET hydrolase LCC, a leaf-branch compost cutinase, and synthesize muconic acid (MA) using the PET hydrolysate. The final product MA and extracellular LCC can be separated from the supernatant of the culture by ultrafiltration, and the latter was used for the next round of PET hydrolysis. A total of 0.50 g MA was produced from 1 g PET in each cycle of the whole biological processes, reaching 68% of the theoretical conversion. This new conceptual scheme for the valorization of PET waste should have advantages over existing PET upcycling schemes and provides new ideas for the utilization of other macromolecular resources that are difficult to decompose, such as lignin.

## 1. Introduction

Polyethylene terephthalate (PET) is one of the most commonly used plastics and widely exists in single-use beverage bottles, textiles, and food packaging [1]. However, when post-consumer PET is poorly managed, it accumulates in the environment, leading to serious environmental pollution and a significant loss of valuable resources [2]. Therefore, an innovative plastic recycling strategy is imperative to achieve the resourceful utilization of PET [3].

PET can be depolymerized to monomers and oligomers by physical and chemical methods, such as pyrolysis, ammonolysis, hydrolysis, and glycolysis [4]. Many chemo-bioprocesses have been developed to synthesize high-value chemicals from the PET waste [5]. Using terephthalate (TPA) produced from PET pyrolysis as the feedstock, Kenny et al. synthesized bioplastic polyhydroxyalkanoates (PHA) employing *Pseudomonas* strains isolated from PET-exposed soil [6], and Kim et al. engineered *Escherichia coli* and *Gluconobacter oxydans* to synthesize higher-value products, such as gallic acid, pyrogallol, muconic acid (MA), vanillic acid, and glycolate [7]. Zhang et al. employed *Taonella mepensis* WT-6 to synthesize bacterial cellulose from PET ammonolysis products [8]. Bis(2-hydroxyethyl) terephthalate (BHET) generated from the glycolysis of PET can be hydrolyzed and converted to value-added compounds, such as protocatechuate, β-ketoadipic acid, and glycollate by engineered strains [9,10]. These studies demonstrated the great potential for bioconversion of the PET hydrolysates to higher-value chemicals.

Recently, many efforts have been devoted to improve the properties of PET hydrolases [11]. *Thermobifida fusca* cutinase (Tfcut2) [12,13], leaf-branch compost cutinase (LCC) [14], and *Ideonella sakaiensis* PETase (*Is*PETase) [15] derived variants with improved thermostability and activity have made enzymatic hydrolysis of PET more efficient and applicable. The most promising variant LCC^ICCG^ can efficiently hydrolyze PET with the TPA productivity up to 16.7 g/(L·h) at 72 °C, providing the possibility of creating a fully biological process for PET recycling [14]. Sadler et al. used semi-purified LCC from *E. coli* BL21(DE3) to hydrolyze PET, and then converted the hydrolysates to vanillin by whole-cell catalysis with another engineered *E. coli* RARE_pVanX [16]. Similarly, Tiso et al. used the purified LCC to hydrolyze PET, and then converted the hydrolysates to PHA by *Pseudomonas umsongensis* GO16 [17]. In our previous study, we have proposed a cocultivation system using the engineered *Yarrowia lipolytica* and *Pseudomonas stutzeri* to achieve the coupling of PET degradation and polyhydroxybutyrate (PHB) production [18]. However, due to the low efficiency of *Is*PETase, it was only able to convert the BHET but not PET into PHB.

Muconic acid (MA) is an important unsaturated dicarboxylic acid, which can be used to produce commodity, new functional resins, pharmaceuticals, and agrochemicals [19]. The current market price of muconic acid is USD 1.5/kg and the global market size is expected to be more than USD 110 million in 2024 [20,21]. Studies have demonstrated MA production from aromatic compounds by directing carbon flux to catechol [22]. The aromatic compound TPA from PET hydrolysate can also be converted to catechol via protocatechuate and, as an example for upcycling, is highly suitable for the biosynthesis of MA. In this study, we designed a new scheme that continuously converts PET to MA using an engineered multifunctional *P. putida* KT2440 that can simultaneously secrete LCC and metabolize PET monomers. PET was first hydrolyzed into TPA and ethylene glycol (EG) employing the LCC crude enzyme from culture supernatant. Subsequently, TPA was converted to MA in the next round of cultivation and another hydrolysate EG was used to support cell growth. MA and LCC in the culture supernatant can be separated by ultrafiltration. The new produced LCC can continue to hydrolyze PET and start a new cycle, allowing continuous production of MA from PET.

## 2. Results and Discussion

### 2.1. Construction of a Multifunctional P. Putida KT2440

**Figure 1:**
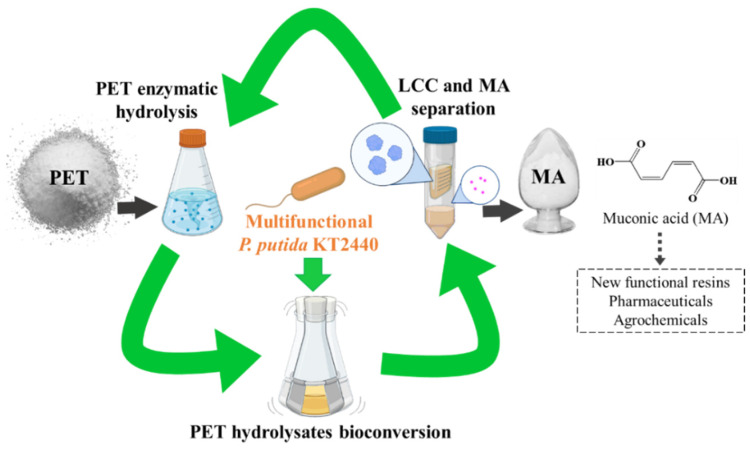
Conceptual scheme for conversion of PET to MA with an engineered multifunctional *Pseudomonas putida*. A leaf-branch compost cutinase (LCC) crude enzyme prepared from the culture supernatant hydrolyzes PET and produces the EG and TPA monomers, which can be directly used as substrates for the following cultivation to produce MA and reproduce LCC. The final product MA can be separated from the LCC crude enzyme by filtration. The remaining concentrated LCC crude enzyme was collected and used for the next round of PET hydrolysis with a stable activity, leading to the continuous conversion of PET to MA.

To achieve the new scheme for upcycling of PET, a multifunctional host that can secrete PET hydrolase and, at the same time, convert the PET hydrolysate TPA and EG to high-value compounds is needed (Figure 1). MA can be applied in the synthesis of new functional resins, pharmaceuticals, and agrochemicals and was selected as the end product [19,22]. *P. putida* KT2440, a soil bacterium that can metabolize a variety of aromatic compounds, was selected as the host chassis [23].

**Figure 2:**
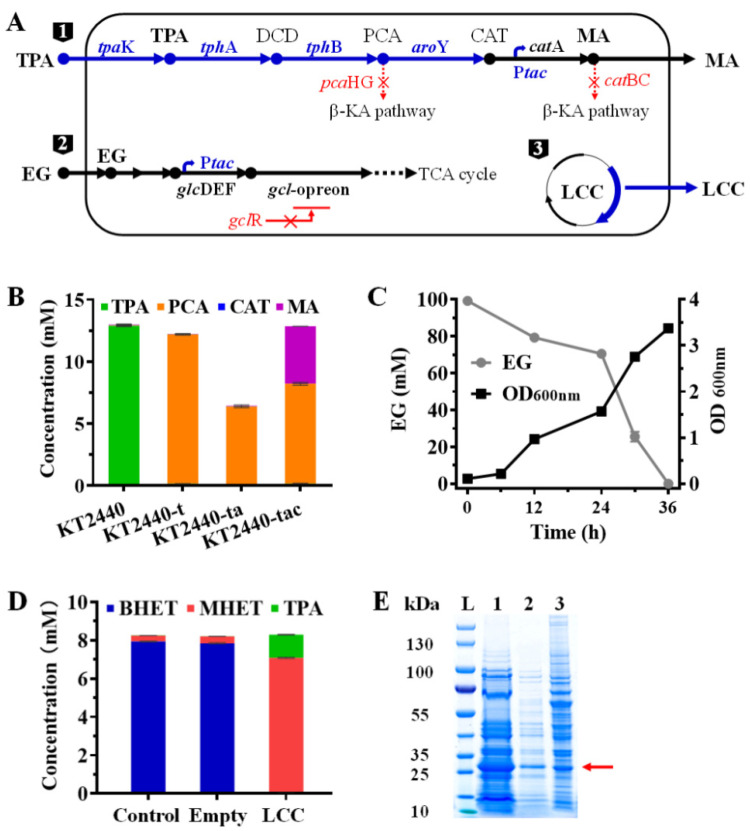
Construction of a multifunctional strain. (**A**), *P. putida* KT2440 as the chassis for metabolic engineering design. 1, engineering the metabolic pathway for converting TPA to MA; 2, enhancing the endogenous EG metabolic pathway; 3, secretory expression of LCC on pBBR1MCS-2. (**B**), Bioconversion of TPA by the derived engineered strains in LB containing TPA. KT2440-t refers to the expression of *tph*-operon by replacing *pca*GH in KT2440; KT2440-ta refers to the further expression of *aro*Y-*ecd*B; KT2440-tac refers to the further deletion of *cat*RBC and the promoter replacement of *cat*A with *tac* promoter. (**C**), Growth and EG metabolism by *P. putida* KT2440-tacRD with the further deletion of *gcl*R and the overexpression of *glc*DEF. (**D**), BHET hydrolysis by LCC crude enzyme from *P. putida* KT2440-tacRDL. Control refers to enzyme-free buffer and empty refers to crude enzyme from strain with an empty vector. (**E**), SDS-PAGE analysis of LCC crude enzyme from *P. putida* KT2440-tacRDL with the further expression of LCC. 1, Concentrated cell-free supernatant (10×); 2, cell-free supernatant; 3, cell lysis sample; TPA, terephthalate; DCD, 1,2-dihydroxy-3,5-cyclohexadiene-1,4-dicarboxylate; PCA, protocatechuate; CAT, catechol; MA, muconic acid; EG, ethylene glycol. TpaK, TPA transporter; TphA, TPA 1,2-dioxygenase; TphB, DCD dehydrogenase; AroY, PCA decarboxylase; CatA, CAT 1,2-dioxygenase; glcDEF, glycolate oxidase; *gcl*-operon, genes involved in glyoxylate carboligase metabolic pathway; gclR, the transcriptional regulator that represses the expression of *gcl*-operon; LCC, leaf-branch compost cutinase. Error bars indicate the standard deviation based on triplicate parallels.

It has been shown that MA can be produced from TPA through a pathway involving the intermediates protocatechuate and catechol by using whole-cell catalyst *E. coli* strain MA-1 [7]. *P. putida* KT2440 was also considered a suitable chassis for the production of MA from TPA, as the part from PCA to MA in the pathway has been proven [24]. In our previous study, we identified a *tph* cluster containing genes encoding the transcriptional regulator (TphR), TPA transporter (TpaK), TPA 1,2-dioxygenase (TphA), and 1,2-dihydroxy-3,5-cyclohexadiene-1,4-dicarboxylate dehydrogenase (TphB) from a TPA degrading *P. Stutzeri* [25]. To utilize TPA and simultaneously block the metabolic branch of the intermediate protocatechuate in *P. putida* KT2440, we replaced protocatechuate 3,4-dioxygenase encoding genes (*pca*HG) with the *tph* cluster identified in *P. stutzeri*. Then, the codon-optimized protocatechuate decarboxylase gene *aro*Y and flavin prenyltransferase gene *ecd*B from *Enterobacter cloacae* were introduced to convert protocatechuate to catechol. EcdB synthesizes a prenylated flavin cofactor required by AroY to enhance the decarboxylase activity [24,26]. Finally, MA biosynthesis pathway was set up by deletion of the downstream metabolic genes *cat*BC [27,28] (Figure 2A). 

To prove the metabolic pathway, the derived engineered strains were cultivated in LB medium containing 2 g/L TPA in a 24-well plate for 24 h. As expected, TPA was almost not consumed by the wild-type *P. putida* KT2440, whereas, in *P. putida* KT2440-t expressing *tph* cluster, TPA was almost fully converted to protocatechuate (Figure 2B). After introducing *aro*Y and *ecd*B, protocatechuate was catalyzed into catechol and further metabolized, resulting in a reduction in protocatechuate to 6.4 mM. When the *cat*RBC genes were further replaced with the *tac* promoter to constitutively express dioxygenase gene *cat*A, the resulting strain *P. putida* KT2440-tac produced 4.63 mM MA from 12.86 mM TPA (Figure 2B), and all of the produced MA was *cis, cis*-MA determined by chromatographic analysis (Figure S8). In the entire MA pathway, the accumulation of the intermediate product protocatechuate was detected, indicating that protocatechuate decarboxylase activity is the catalytic bottleneck [29,30,31]. However, even so, the final engineered strain *P. putida* KT2440-tac has been able to convert TPA into MA. 

EG, another hydrolysis product of PET, can be sequentially oxidized to glyoxylate, and then supports the growth of *P. putida* KT2440 via the glyoxylate carboligase (*gcl*) pathway (Figure 2A) [32]. However, wild-type *P. putida* KT2440 cannot grow on EG because the *gcl*-operon was repressed by a specific transcriptional regulator (*gcl*R) [33]. To make *P. putida* KT2440 grow on EG, we knocked out *gcl*R and overexpressed glycolate oxidase gene (*glc*DEF) via the strong, constitutive *tac* promoter, as described by Werner [10]. The resulting *P. putida* KT2440-tacRD (Δ*pca*HG::*tph*::*aro*Y:*ecd*B Δ*cat*RBC::P*tac*:*cat*A Δ*gcl*R::P*tac*:*glc*DEF) strain was confirmed to grow on EG (Figure 2C).

To further achieve the production of PET hydrolase in *P. putida* KT2440-tacRD, LCC was expressed on pBBR1MCS-2 driven by *lac* promoter (Figure 2A). We found that the extracellular LCC was comparable whether with or without the native signal peptide (Figure S1). The transfer of LCC from the cytoplasm to the outside of the cell was probably attributed to the activity of phospholipid hydrolyzing, resulting in membrane permeation [34,35]. The 27.8 kDa protein band representing LCC was clearly shown on the SDS-PAGE (Figure 2E). To verify the activity of LCC, culture supernatant was collected to catalyze BHET emulsion hydrolysis. It was proven by HPLC that BHET was almost completely hydrolyzed to MHET and TPA within 1 h by crude enzyme from *P. putida* KT2440-tacRDL expressing LCC. In comparison, enzyme-free phosphate buffer (Control) and the crude enzyme from *P. putida* KT2440-tacRD containing pBBR1MCS-2 without expressing LCC (Empty) cannot hydrolyze BHET (Figure 2D).

Hereto, *P. putida* KT2440-tacRDL (Δ*pca*HG::*tph*::*aro*Y:*ecd*B Δ*cat*RBC::P*tac:cat*A Δ*gcl*R P*tac*:*glc*DEF) (pBBR-LCC) achieved the expectation, namely secreting PET hydrolase LCC and simultaneous production of MA using PET hydrolysates.

### 2.2. Enzymatic Hydrolysis of PET Using LCC Crude Enzyme

**Figure 3:**
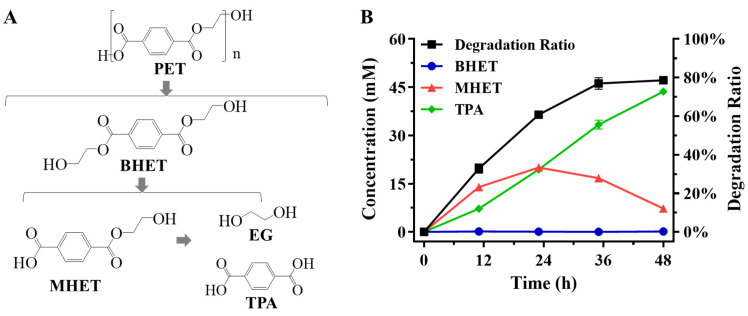
PET enzymatic hydrolysis using the LCC crude enzyme produced by *P. putida* KT2440-tacRDL. (**A**), The process of enzymatic hydrolysis of PET. (**B**), The degradation curves of PET enzymatic hydrolysis. The experiments were performed in duplicate.

A total of 50 mL culture supernatant of *P. putida* KT2440-tacRDL was concentrated to 1 mL LCC crude enzyme by filtration with a 10 kDa ultrafilter. The total protein concentration was determined to be 5 mg/mL with the folin phenol reagent [36], and the content of LCC was determined to be 38.70% by image analysis using ImageJ [37] (Figure S2). As a proof, 0.125 g amorphous PET powder and 1 mL LCC crude enzyme (~15 milligrams of LCC per gram of PET) was added to 100 mM phosphate buffer to a total volume of 10 mL. The enzyme was considered to be sufficient relative to the substrate, because it was shown by Tournier that a ratio of 3 milligrams of enzyme per gram of PET appeared to maximize the depolymerization [14]. The catalytic reaction was carried out at 72 °C in a water bath shaker. During the hydrolysis of PET, there was little accumulation of BHET with the concentration less than 0.23 mM, while the amount of MHET increased first and then decreased to produce TPA (Figure 3). The TPA productivity over the whole reaction was about 0.15 g/(L·h) in a 10 mL scale reaction system under the experimental conditions. The reaction was stopped at 48 h as the degradation ratio of PET reached 79% and no longer increased (Figure 3B). The final concentration of TPA reached 43.66 mM (Figure 3B), that is, at least 0.58 g TPA can be produced from 1 g PET in a hydrolysis process catalyzed by LCC crude enzyme under the above conditions. The results demonstrated the feasibility of directly catalyzing PET hydrolysis using LCC crude enzyme produced by *P. putida* KT2440-tacRDL.

### 2.3. Bioconversion of the PET Hydrolysates into MA by the Engineered Multifunctional Strain

To convert PET hydrolysate into MA, we first evaluated the conversion of TPA to MA by *P. putida* KT2440-tacRDL, with EG as the sole carbon source. The cultivation was performed in mineral medium containing simulated PET hydrolysates (20 mM EG and TPA) at 30 °C. However, the OD_600nm_ only reached about 1.0, and 6.71 mM TPA was converted when EG was completely consumed. The product was mainly PCA, and only 0.70 mM MA was accumulated (Figure S3). It indicated that additional nutrients need to be added to the PET hydrolysate to provide sufficient energy for cell growth and TPA conversion.

Therefore, the bioconversion was evaluated in LB containing 20 mM simulated PET hydrolysates at 30 °C and 37 °C, respectively. The cell growth and the bioconversion of TPA were significantly improved when cultivated in LB (Figure S4). TPA was almost completely converted in 36 h; however, the subsequent conversion of PCA slowed down with cell growth arrest at 18 h, leading to the residue of PCA (Figure S4). Interestingly, the residual PCA at 37 °C was less than that at 30 °C, which led to a higher yield of MA at 37 °C (14.09 mM, the yield at 30 °C was 7.59 mM, the maximum theoretical yield was is the actual detected initial concentration of TPA, about 17.00 mM) (Figure S4). We speculate that *aro*Y from *E. cloacae* may have higher PCA decarboxylase activity at 37 °C. We also found that the yield of LCC at 37 °C was higher than that at 30 °C. Therefore, the subsequent experiments were all carried out at 37 °C. To continue the conversion of PCA, we supplemented glucose at 24 h to maintain the cell growth. Under the conditions, TPA was completely converted to MA within 36 h without the accumulation of intermediates (Figure S5). 

Finally, the bioconversion of actual PET hydrolysates was performed under the optimized conditions. PET powder was hydrolyzed by LCC produced from *P. putida* KT2440-tacRDL to generate the actual hydrolysates, which were mixed with 2× LB in equal volumes. The initial concentrations of TPA and EG in the medium were determined to be 31.86 mM and 29.83 mM, respectively. Glucose was supplemented to maintain the cell growth. The cultivation lasted for 60 h until TPA was completely converted to MA without the accumulation of intermediates. The final concentration of MA reached 39.54 mM, which was higher than the initial concentration of TPA (31.86 mM). It may be caused by the volatilization of the medium, because the final volume of the culture medium (35.0 mL) was less than the theoretical residual volume (42.8 mL) calculated from sampling and feeding volume. According to this ratio, the final concentration of MA should be 32.33 mM, which is slightly higher than 31.86 mM. The synthesis of MA from glucose requires the expression of several heterologous genes, such as 3-deoxy-D-arabinoheptulosonate 7-phosphate synthase necessary for entry into the shikimate pathway [38], which are not present in the engineering strain *P. putida* KT2440-tacRDL. Therefore, we believe that all of the MA is converted from TPA and not the glucose. The still slightly higher conversion of TPA to MA (101.48%) is probably due to the experimental error. The productivity of MA reached 0.54 mmol/(L·h), which was higher than the rate of producing MA from glucose and lignin-derived aromatic compounds by engineered *P. putida* KT2440-CJ083 in shake flask experiments [0.28 mmol/(L·h)] [24] and the rate of producing MA from PET hydrolysates by engineered *E. coli* MA-1 in whole-cell catalysis experiments [0.45 mmol/(L·h)] [7]. 

**Figure 4:**
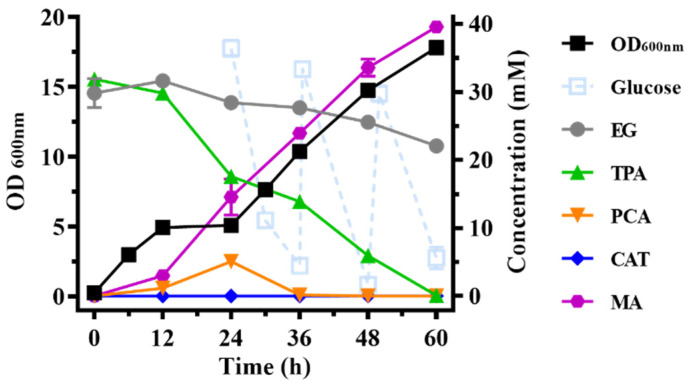
Bioconversion of the PET hydrolysate to produce MA by *P. putida* KT2440-tacRDL. TPA, terephthalate; PCA, protocatechuate; CAT, catechol; MA, muconic acid; EG, ethylene glycol. The experiments were performed in duplicate.

The metabolism of EG was significantly inhibited when the cultivation was performed in LB medium (Figure 4 and Figures S4 and S5). A similar phenomenon also appeared in Werner’s study, when producing β-ketoadipic acid from BHET by engineered *P. putida* KT2440 [10]. They considered that part of the EG catabolic pathway is repressed by β-ketoadipic acid rather than glucose. However, β-ketoadipic acid was not involved in our study. Therefore, there may be a more complex regulatory mechanism on the metabolism of EG in *P. putida* KT2440.

In conclusion, PET monomer TPA can be converted to MA at 100% molar yield by *P. putida* KT2440-tacRDL. According to the molecular weight calculation, 0.50 g MA can be produced per gram PET in one cycle of valorization, reaching 68% of the theoretical conversion.

### 2.4. Separation of LCC from MA in Culture Supernatant for Continuous PET Valorization

**Figure 5:**
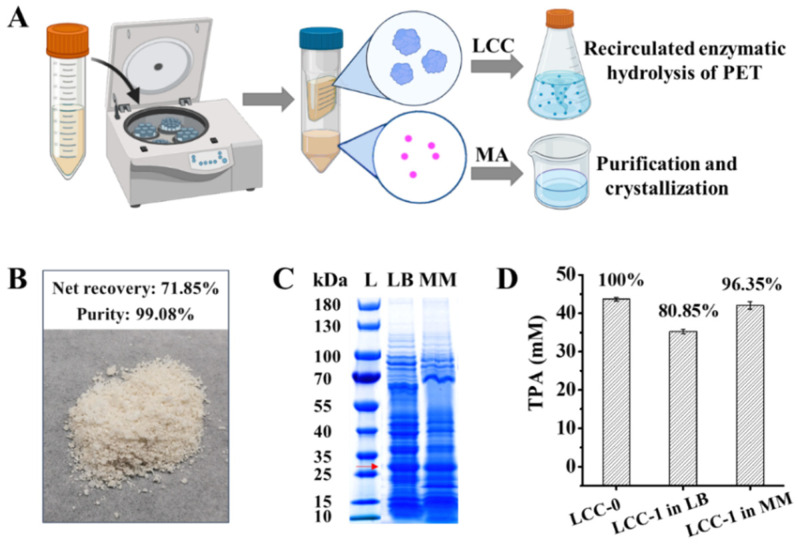
Separation of LCC from MA and used for the new round of hydrolysis of PET. (**A**), The process for separating the end product MA and the reproduced LCC; (**B**), recovery rate and purity of the obtained MA powder; (**C**), SDS-PAGE analysis of the crude enzyme produced when the bioconversion of PET hydrolysates occurred concurrently in LB or mineral medium (MM). There is a clear thickened band at the size of LCC (approximately 27.8 kDa); (**D**), activity comparison of LCC crude enzyme form different resources. LCC-0, crude enzyme produced from LB without bioconversion of PET hydrolysates; LCC-1 in LB, crude enzyme produced from LB when the bioconversion occurred concurrently; LCC-1 in MM, crude enzyme produced from MM when the bioconversion occurred concurrently. Error bars indicate the standard deviation based on duplicate parallels.

During the production of MA, a new batch of PET hydrolase LCC was produced by *P. putida* KT2440-tacRDL at the same time. Newly produced LCC should be separated from the value-added product MA and used for the next round of hydrolysis of PET to achieve a continuous PET valorization. To achieve this purpose, culture supernatant containing extracellular LCC and MA was collected by centrifugation at 12,000 rpm to remove cell debris. Then, the supernatant was filtrated with a 10 kDa ultrafilter, wherein LCC was in the concentrate and MA was in the filtrate (Figure 5A). MA was extracted from cultures with different substrate concentrations by crystallization and purification as described in methods. The recovery reached 71.85 % and the purity was determined by HPLC to exceed 99% (Figure 5B, Table S2).

As for PET hydrolase LCC, SDS-PAGE showed a protein band consistent with the size of LCC, indicating the cultivation on the PET hydrolysates did not affect the expression of LCC (Figure 5C). The new produced LCC crude enzyme from LB containing PET hydrolysates was used for the new round of PET hydrolysis to achieve the continuous PET valorization. In total, 35.30 mM TPA was produced within 48 h, equivalent to 81% of the LCC activity produced in LB without PET hydrolysates (Figure 5D and Figure S7A). In addition, to reduce the cost, we also evaluated the conversion of PET hydrolysates and reproduction of LCC in low-cost mineral medium with glucose as the supplemented carbon source. The conversion rate of TPA to MA in mineral medium was lower than that in LB (Figure S6). However, it was surprising that 42.06 mM TPA was produced within 48 h from PET hydrolysis catalyzed by the LCC crude enzyme prepared from the mineral medium, which is equivalent to 96% of the LCC activity produced in LB without PET hydrolysates (Figure 5D and Figure S7B). These results indicated that the new produced LCC was stable in yield and activity during the bioconversion of PET hydrolysates and can be sustained for the new round of hydrolysis of PET.

## 3. Materials and Methods

### 3.1. Strains and Cultivation

*E. coli* DH5α used for plasmid construction and clone was cultivated in LB (10 g/L tryptone, 5 g/L yeast extract, and 10 g/L NaCl) at 37 °C and 220 rpm. Primary and conventional cultivation of *P. putida* KT2440 and derived strains was performed in LB at 30 °C and 220 rpm. Mineral medium (MM) used in this study contains 34.74 g/L Na_2_HPO_4_·12H_2_O, 0.408 g/L KH_2_PO_4_, 2 g/L NH_4_Cl, 1 g/L NaCl, 2 mM MgSO_4_, 0.1 mM CaCl_2_, and 1 mL/L trace element solution (TES). TES contains 50 g/L Na_2_EDTA, 20 g/L ZnSO_4_·7H_2_O, 5.5 g/L CaCl_2_, 5 g/L MnCl_2_·4H_2_O, 1.0 g/L (NH_4_)_6_Mo_7_O_24_·4H_2_O, 5.0 g/L FeSO_4_·7H_2_O, CuSO_4_·5H_2_O 1.5 g/L, and 1.61 g/L CoCl_2_·5H_2_O. If necessary, kanamycin antibiotic was added as the working concentration of 25 mg/L. The content of agar in the solid medium was 20%.

### 3.2. Plasmid Construction and Strain Engineering

Codon-optimized *aro*Y (GenBank: ADF61496), *ecd*B (GenBank: ADF63617) [24], and LCC^ICCG^ (hereafter referred to as LCC) [14] genes were synthesized by GeneralBio, China. The *tph* operon genes *tph*R (GenBank: QTF59206), *tph*A1 (GenBank: QTF59202), *tph*A2 (GenBank: QTF59205), *tph*A3 (GenBank: QTF59204), *tph*B (GenBank: QTF59203), and *tpa*K (GenBank: QTF59201) were cloned from a TPA degrading *P. stutzeri* isolated from PET waste in our previous study [25]. Polymerase chain reaction (PCR) was performed with Phanta Max Super-Fidelity DNA Polymerase (Vazyme). Oligonucleotides used in this study were synthesized by TsingKe, China, and are shown in Table S1. The plasmid backbone and DNA fragments were assembled using MultiF Seamless Assembly Mix (ABclonal) according to the manufacturer’s instructions. The assembled products were directly transformed into *E. coli* DH5α chemically competent cell (TsingKe, China) for plasmid maintenance. Colony PCR was performed with 2× Taq Plus Master Mix II (Dye Plus) (Vazyme) and plasmid inserts were confirmed with Sanger sequencing performed by TsingKe, China. The gene replacement and deletion of *P. putida* KT2400 were performed via two-step recombination using the vector pK18mobsacB. Initial recombination into the chromosome was selected based on kanamycin resistance gene on LB plates containing 25 mg/L kanamycin, and the second recombination was selected based on sucrose lethal gene (*sac*B) on LB plates containing 20% sucrose. The final correct strains were confirmed by colony PCR and Sanger sequencing. The detailed information of plasmids and strains are shown in Table 1.

### 3.3. Preparatin of LCC Crude Enzyme

*P. putida* KT2400-tacRDL was inoculated from the overnight cultivated seeds into 50 mL LB containing 25 mg/L kanamycin, and cultivated at 37 °C and 220 rpm for 24 h. This expression system is constitutive due to the absence of *lac*I both in the used plasmid and *P. putida* KT2400 [23,41], so it does not need the addition of isopropyl β-d-1-thiogalactopyranoside (IPTG). Cells were removed by centrifugation at 12,000 rpm and the cell-free supernatant was filtered with a 10 kDa ultrafilter (Millipore, Burlington, MA, USA) and concentrated to obtain 1 mL LCC crude enzyme. Cells were disrupted with an automatic sample rapid grinder (Jingxin Technology, Shanghai, China) [42] and analyzed by sodium dodecyl sulfate polyacrylamide gel electrophoresis (SDS-PAGE), as with the cell-free supernatant, before and after concentration. Total protein concentration of the crude enzyme was determined with the folin phenol reagent [36], and the content of LCC was determined by image analysis using online ImageJ [37] (https://cnij.imjoy.io/ (accessed on 24 February 2022)).

### 3.4. Enzyme Assays on Bis(2-Hydroxyethyl) Terephthalate (BHET)

The activity of extracellular LCC was preliminarily detected with BHET as substrate. In total, 0.4 g BHET particles (Sigma) were added to 100 mL 100 mM phosphate buffer (pH 8.0) to prepare the emulsion by ultrasonic pretreatment for 2 min and magnetic stirring for 30 min. The reaction system contained 300 μL 100 mM phosphate buffer, 200 μL LCC crude enzyme, and 500 μL BHET emulsion and reacted at 40 °C for 1 h. The reaction was stopped by mixing with an equal volume of acetonitrile. The samples were analyzed using high-performance liquid chromatography (HPLC) after centrifugation and filtration. Three parallel experiments were carried out in each assay.

### 3.5. Enzymatic Hydrolysis of PET

Amorphous PET film (Goodfellow) was micronized under the condition of liquid nitrogen freezing. The particle size of the PET powder was limited to 425 μm by a 35-mesh screen. A total of 0.125 g PET powder and 1 mL LCC crude enzyme prepared from 50 mL culture supernatant were added to 100 mM phosphate buffer (pH 8.0) to a total volume of 10 mL. The catalytic reaction was carried out at 72 °C and 150 rpm in a water bath shaker (Yijing Technology, Beijing, China). Samples were taken every 12 h and the pH of the reaction system was adjusted to 8.0 with NaOH. The sample was treated with an equal volume of acetonitrile to stop the reaction and analyzed using HPLC after centrifugation and filtration. Two parallel experiments were carried out in each assay.

The weight loss of PET was proved in a good quantitative agreement with the determined sum of aromatic products released [43]. Therefore, in this study, the estimated degradation ratio of PET was calculated from the sum of TPA, MHET, and BHET measured. The equation used to calculate the degradation ratio of PET was the following:

PET degradation ratio=CTPAMWTPA+CMHETMWMHET+CBHETMWBHETWPETMWPETV×100%

where C_TPA_ (g/L), C_MHET_ (g/L) and C_BHET_ (g/L) refer to the concentration of TPA, MHET and BHET measured at a specific reaction time; W_PET_ (g) and V (L) refer to initial weight of PET and the reaction volume, respectively. MW_PET_, MW_TPA_, MW_MHET_, and MW_BHET_ are the molecular weights of PET repeating unit (192 g/mol), TPA (166 g/mol), MHET (210 g/mol), and BHET (254 g/mol), respectively.

### 3.6. Well Plate Cultivation for the Bioconversion of TPA

*P. putida* KT2440 and the derived strains KT2440-t, KT2440-ta, and KT2440-tac were pre-cultivated in LB at 30 °C for 12 h. Then, 0.1 mL of the prepared cultures was inoculated into 1 mL of LB containing 2 g/L TPA in a 24-well plate at 30 °C and 400 rpm for 24 h. The cultures were transferred to 1.5 mL centrifuge tubes and centrifuged at 12,000 rpm for 5min. The supernatants were filtered with a 0.22 filter membrane and analyzed by HPLC.

### 3.7. Shake Flask Cultivation on the PET Hydrolysates

Cultivation on PET hydrolysates was performed in 50 mL MM or LB medium containing simulated or actual PET hydrolysates in a shake flask. Simulated PET hydrolysates were prepared with 20 mM TPA and EG. Actual PET hydrolysates were prepared from PET enzymatic hydrolysis by LCC produced by *P. putida* KT2440-tacRDL. The hydrolysates were centrifuged and mixed with 2× LB in equal volume, and then sterilized. Glucose was appropriately supplemented to maintain cell growth during the cultivation.

### 3.8. Separation of LCC and MA

Cells were removed from the culture broth by centrifugation at 12,000 rpm to obtain the cell-free supernatant containing extracellular LCC and MA. LCC and MA were separated by filtration with 10 kDa ultrafilter (Millipore), wherein LCC was in the concentrate and MA was in the filtrate. Concentrated LCC crude enzyme was used again for PET hydrolysis. MA was separated and purified from the filtrate according to the method reported by Beckham [44]. The specific steps include: 1, remove color compounds with 5 wt% activated carbon powder; 2, crystallize at pH 2 and 5 °C; 3, redissolve the crystals in ethanol and filtered to remove insoluble salts. Finally, the recovered MA was dried and the purity determined by HPLC.

### 3.9. Substrate and Product Analysis

Glucose was measured using an SBA-40C biosensor (developed by Biology Institute of Shandong Academy of Sciences) equipped with glucose oxidase immobilized on membranes [39].

EG was detected on SHIMADZU LC-20A HPLC system equipped with a refractive index detector and a Bio-Rad Aminex HPX-87H column (7.8 × 300 mm) [18]. The column temperature was 65 °C. The mobile phase was 5 mM H_2_SO_4_ and the flow rate was 0.6 mL/min.

Enzymatic hydrolysates, including BHET, MHET, and TPA (Figure S9), were detected by a photodiode array detector at 240 nm using SHIMADZU LC-20A HPLC system equipped with an Agilent ZORBAX Extend-C18 column (4.6 × 150 mm), as previously [18]. The mobile phase was a solution containing 0.1% (*v/v*) trifluoroacetic acid and 20% (*v/v*) acetonitrile. The flow rate and column temperature were set to 0.8 mL/min and 40 °C, respectively.

Products in the culture broth, including TPA, protocatechuate, catechol, and MA, were detected by a photodiode array detector at 230 nm using SHIMADZU LC-20A HPLC system equipped with a Discovery HS C18 (4.6 × 250 mm) column. The mobile phase was a solution containing 0.1% (*v/v*) trifluoroacetic acid and 10% (*v/v*) acetonitrile. The flow rate and column temperature were set to 0.8 mL/min and 40 °C, respectively.

## 4. Conclusions

In this study, we designed a new conceptual scheme for the continuous valorization of PET to MA. The well-designed multifunctional strain is capable of converting TPA to MA accompanied by the production of extracellular PET hydrolase. This is more simplified and cost-effective than the process of using recombinant *E. coli* to produce enzymes and then using another strain for PET hydrolysate conversion. By optimizing the entire process, we achieved an efficient bioconversion of PET into MA (0.50 g MA/g PET). In order to make a practical application, each section of the process requires scale-up experiments and assesses the feasibility and cost. In addition, since efficient enzymatic hydrolysis of PET using LCC requires the high reaction temperature (72 °C) but *P. putida* KT2440, the chassis chosen in this study, is a mesophilic strain, the enzymatic hydrolysis of PET and the bioconversion of PET hydrolysate had to be separated into two independent processes. Recently, a new variant of *Is*PETase was reported to depolymerize PET efficiently at 50 °C [45], which increases the possibility of realizing the one-pot biological valorization of PET, because it is easier for strains to tolerate 50 °C than 72 °C. As a paradigm, this study aims to provide an illustration for the eventual realization of the one-pot biological valorization of PET.

## Figures and Tables

**Table 1:** Strains and plasmids used in this study.

Strains and Plasmids	Relevant Properties	Sources
pK18mobsacB	Allelic exchange vector, *ori*ColE1 Mob^+^, *sac*B, Km^r^	Lab Stock [39]
pBBR1MCS-2	Protein expression vector, pBBR1 replicon, Mob^+^, Km^r^	Lab Stock [40]
pBBR-LCC	LCC expression driven by *lac* promoter on pBBR1MCS-2	This study
*E. coli* DH5α	F– φ80*lac*Z∆M15 ∆(*lac*ZYA-argF)U169 *deo*R *rec*A1 *end*A1 *hsd*R17(rK–, mK+) *pho*A *sup*E44 λ– *thi*-1 *gyr*A96 *rel*A1, used for plasmids construction and clone.	Lab Stock
*P. putida* KT2440	Wild-type	Lab Stock
*P. putida* KT2440-t	*P. putida* KT2440 (Δ*pca*HG::*tph*), genomic replacement of *pca*HG with tph cluster (*tph*RA2A3A1BK).	This study
*P. putida* KT2440-ta	*P. putida* KT2440 (Δ*pca*HG::*tph*::*aro*Y:*ecd*B), additional insertion of codon-optimized *aro*Y and *ecd*B follow tph cluster.	This study
*P. putida* KT2440-tac	*P. putida* KT2440 (Δ*pca*HG::*tph*::*aro*Y:*ecd*B Δ*cat*RBC::P*tac*:*cat*A), additional genomic replacement of *cat*RBC with *tac* promoter, which enabled constitutive expression of *cat*A	This study
*P. putida* KT2440-tacR	*P. putida* KT2440 (Δ*pca*HG::*tph*::*aro*Y:*ecd*B Δ*cat*RBC::P*tac*:*cat*A Δ*gcl*R), additional *gcl*R deletion.	This study
*P. putida* KT2440-tacRD	*P. putida* KT2440 (Δ*pca*HG::*tph*::*aro*Y:*ecd*B Δ*cat*RBC::P*tac*:*cat*A Δ*gcl*R::P*tac*:*glc*DEF), additional promoter replacement of *glc*DEF wih *tac* promoter.	This study
*P. putida* KT2440-tacRDL	*P. putida* KT2440 (Δ*pca*HG::*tph*::*aro*Y:*ecd*B Δ*cat*RBC::P*tac*:*cat*A Δ*gcl*R::P*tac*:*glc*DEF) (pBBR-LCC), additional LCC expression on pBBR1MCS-2	This study

## Data Availability

Any data relating to this study will be made available upon reasonable requests.

## Supplementary Materials

The following supporting information can be downloaded at: https://www.mdpi.com/article/10.3390/ijms231910997/s1.
