# Supplementary material for: Valorization of Polyethylene Terephthalate to Muconic Acid by Engineering Pseudomonas Putida
Source: Int J Mol Sci. 2022 Sep 20;23(19):10997. doi: 10.3390/ijms231910997 (PMC9569715; doi:10.3390/ijms231910997)
Supplement: Supplementary file 1 [file ijms-23-10997-s001.zip › ijms-1919616-supplementary.pdf]

Supplementary materials

Valorization of polyethylene terephthalate to muconic acid by engineering *Pseudomonas putida*

Pan Liu, Yi Zheng, Yingbo Yuan, Tong Zhang, Qingbin Li, Quanfeng Liang, Tianyuan Su*, Qingsheng Qi*

State Key Laboratory of Microbial Technology, Shandong University, Qingdao 266237, China

*Correspondence: sutianyuan@sdu.edu.cn (Tianyuan Su); qiqingsheng@sdu.edu.cn (Qingsheng Qi); Tel & Fax: +86-532-58632580

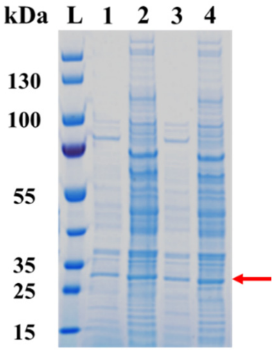

Figure S1. SDS-PAGE analysis when expressing LCC with and without the native signal peptide at 30 °C. 1, extracellular proteins and 2, cytoplasm proteins when expressing LCC without signal peptide; 3, extracellular proteins and 4, cytoplasm proteins when expressing LCC with signal peptide.

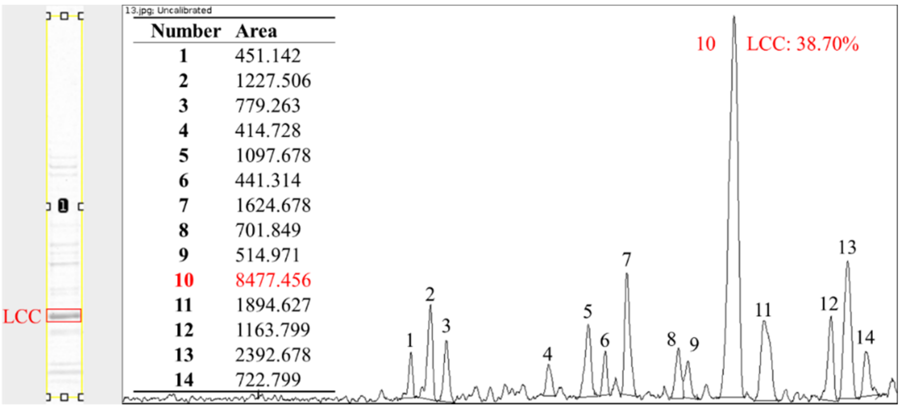

Figure S2. Densitometry band quantification of LCC in the crude enzyme from *P. putida* KT2440-tacRDL by ImageJ [1]. The content of LCC in the crude enzyme was determined to be 38.70% of total protein.

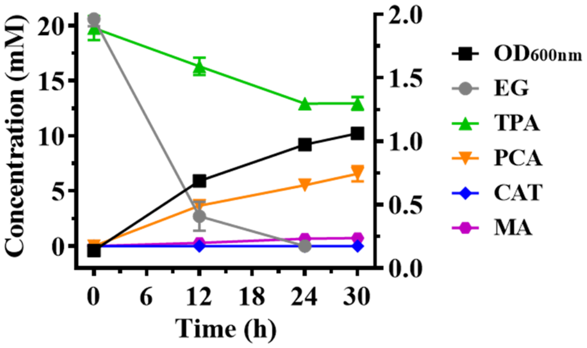

Figure S3. Bioconversion of 20 mM simulated PET hydrolysates into MA in mineral medium without supplemented carbon source by *P. putida* KT2440-tacRDL at 30 °C. EG, ethylene glycol; TPA, terephthalate; PCA, protocatechuate; CAT, catechol; MA, muconic acid. The experiments were performed in duplicate.

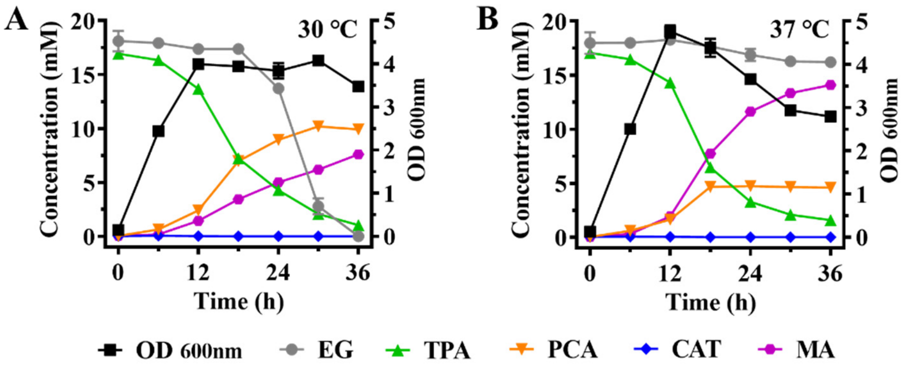

Figure S4. Bioconversion of 20 mM simulated PET hydrolysates into MA in LB by *P. putida* KT2440-tacRDL at different temperature. A, at 30 °C; B, at 37 °C. EG, ethylene glycol; TPA, terephthalate; PCA, protocatechuate; CAT, catechol; MA, muconic acid. The experiments were performed in triplicate.

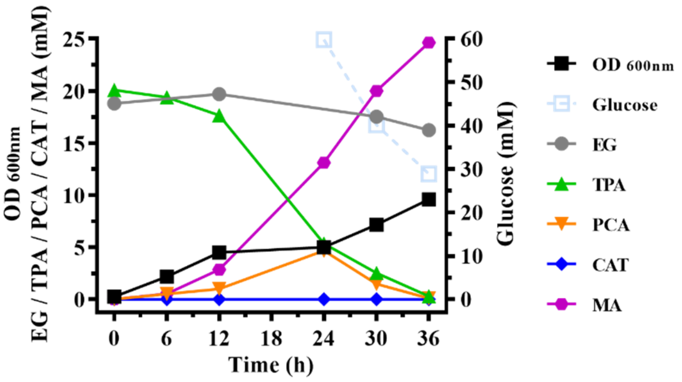

Figure S5. Bioconversion of 20 mM simulated PET hydrolysates into MA in LB with supplemented glucose by *P. putida* KT2440-tacRDL at 37 °C. The experiments were performed in triplicate.

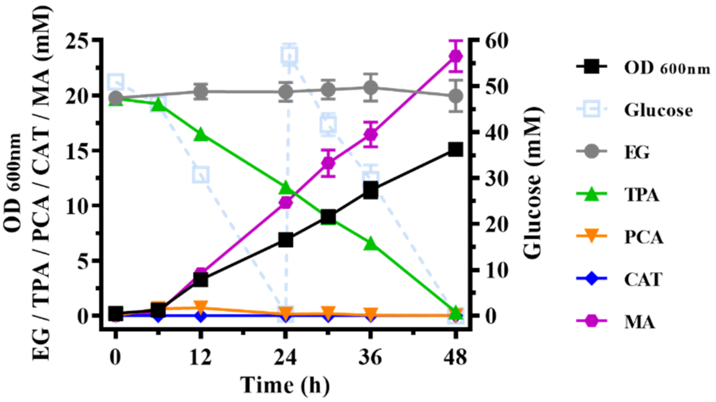

Figure S6. Bioconversion of 20 mM simulated PET hydrolysates into MA in mineral medium with supplemented glucose by *P. putida* KT2440-tacRDL at 37 °C. The experiments were performed in triplicate.

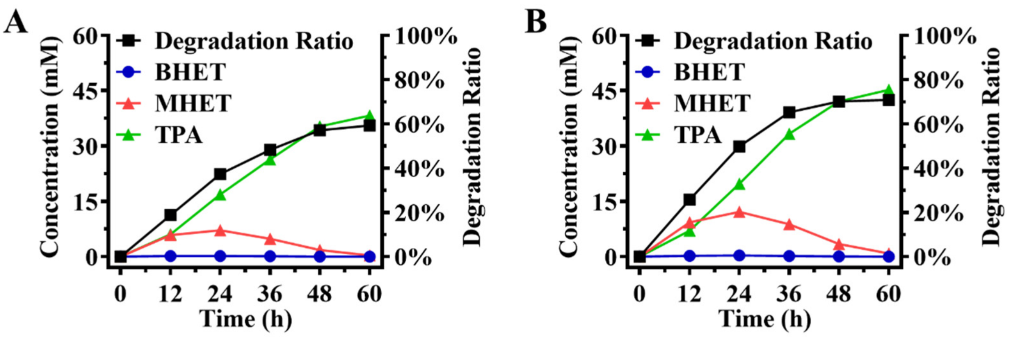

Figure S7. PET hydrolysis catalyzed by LCC crude enzyme produced during the bioconversion of PET hydrolysates in different medium. A, crude enzyme was produced from LB containing PET hydrolysates; B, crude enzyme was produced from mineral medium (MM) containing PET hydrolysates. The experiments were performed in duplicate.

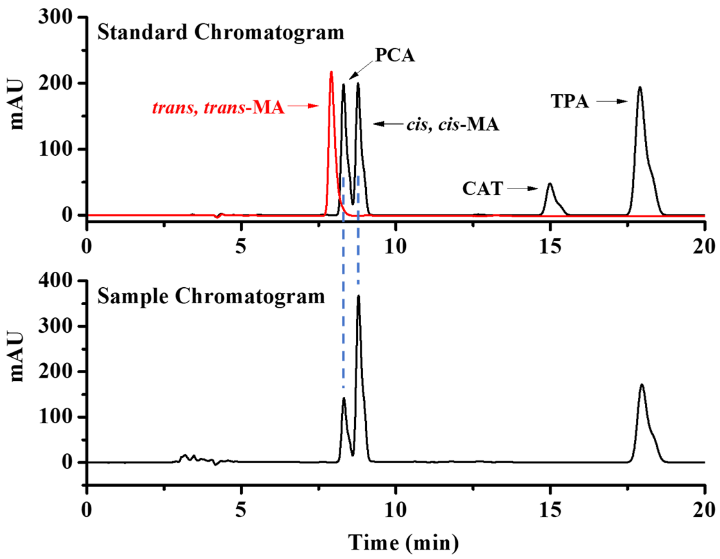

Figure S8. Liquid chromatogram of the bioconversion products and corresponding standards detected.

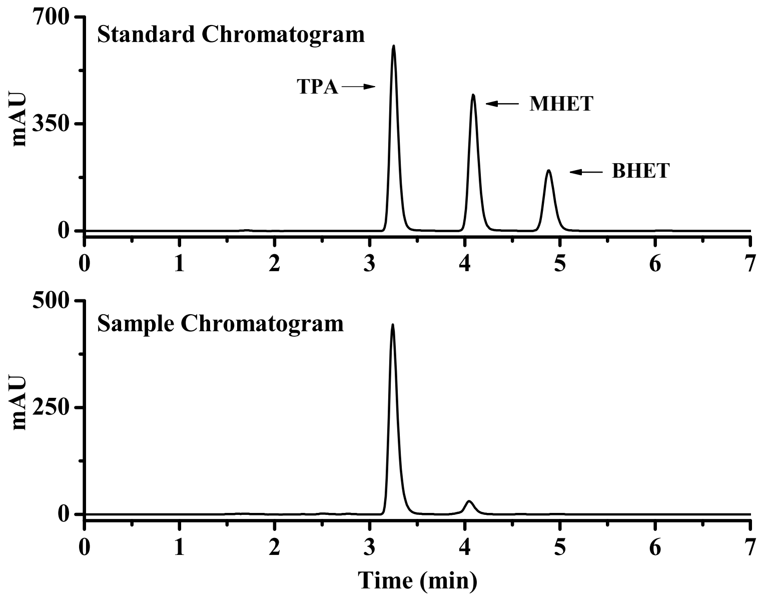

Figure S9. Liquid chromatogram of PET enzymatic hydrolysates and corresponding standards.

Table S1. Oligonucleotides used in this study.

Primers	Sequences (5′–3′)	Purposes
pk18F	GGATCCTCTAGAGTCGACCT	To amplify the plasmid backbone of pK18mobsacB for genes deletion or replacement.
pk18R	AATTGCGTTGCGCTCACTGC	
pcaHGarm1F	AGCGGGCAGTGAGCGCAACGCAATTgatgcgatgatcacgatgtg	To amplify the upstream homology region of pcaHG for the replacement with tph cluster. The primer pairs each contain homologous sequences with pK18mobsacB backbone and tph cluster for Gibson assembly.
pcaHGarm1R	tgctttgctcgggtgcatgctgtaacaacgatctgcgtacaccga	
pcaHGarm2F	ttatggcaacagcaataaggtttaatggaattgtgagaacgcctg	To amplify the downstream homology region of pcaHG for the replacement with tph cluster. The primer pairs each contain homologous sequences with tph cluster and pK18mobsacB backbone for Gibson assembly.
pcaHGarm2R	CCTGCAGGTCGACTCTAGAGGATCCtctgcacatggtgaccgatt	
tphF	ttacagcatgcacccgagca	To amplify tph cluster, which contains tphR, tphA2, tphA1, tphA3, tphB, tpaK.
tphR	ttaaaccttattgctgttgcc	
tpharm1F	GCAGTGAGCGCAACGCAATTttgatgctggttcccgtcct	To amplify the upstream homology region for the insertion of aroY:ecdB follow tph cluster. Primer tpharm1F contains homologous sequences with pK18mobsacB backbone for Gibson assembly.
tpharm1R	acctgccggaaactgcaagt	

tpharm2F	gaacaacgacggcatctgacgcgcgtgatgaatattgaa	Tpharm2F and pcaHGarm2R were used to amplify the downstream homology region for the insertion of aroY:ecdB follow tph cluster. The primer pairs each contain homologous sequences with tph cluster and pK18mobsacB backbone for Gibson assembly.
aroY-ecdBF	cttgcagtttccggcaggtGATCTGAGCTGTTGACAATT	To amplify codon optimized aroY-ecdB. Primer aroY-ecdBF contains homologous sequences with upstream homology region for Gibson assembly.
aroY-ecdBR	tcagatgccgtcgttgttct	
catarm1F	AGCGGGCAGTGAGCGCAACGCAATTgtggagtcgatgatgatcag	To amplify the upstream homology region of catRBC for the replacement with tac promoter. Primer catarm1F contains homologous sequence with pK18mobsacB backbone for Gibson assembly. Primer catarm1R contains tac promoter sequences, which is homologous to primer catarm2F for Gibson assembly.
catarm1R	ccacacattatacgagccgatgattaattgtcaacagccgctgtatagccctgccctat	
catarm2F	aatcatcggctcgtataatgtgtggaatcagcttgcacaacaatacgaggtaagcacg	To amplify the downstream homology region of catRBC for the replacement with tac promoter. Primer catarm2F contains tac promoter sequence, which is homologous to primer catarm1R for Gibson assembly. Primer catarm2R contains homologous sequence with pK18mobsacB backbone for Gibson assembly.
catarm2R	CCTGCAGGTCGACTCTAGAGGATCCcgagatgaagaagtgcacgt	
gclRarm1F	GCAGTGAGCGCAACGCAATTccgatagcagcaccgatca	To amplify the upstream homology region of gclR for its deletion. The primer pairs each contain homologous sequences with pK18mobsacB backbone and downstream homology region for Gibson assembly.
gclRarm1R	gtgcctcgagagatcgtttgcctgcgtgatcg	
gclRarm2F	caggcaaacgatctctcgaggcacgaagagaa	To amplify the downstream homology region of gclR for its deletion. The primer pairs each contain homologous sequences with upstream homology region and pK18mobsacB backbone for Gibson assembly.
gclRarm2R	GCAGGTCGACTCTAGAGGATCCcgatgatgcctgcagtc	
glcDEFarm1F	AGTGAGCGCAACGCAATTtcaccccagccatccata	To amplify the upstream homology region for the promoter replacement of glcDEF. Primer glcDEFarm1F contains homologous sequence with pK18mobsacB backbone for Gibson assembly. Primer glcDEFarm1R contains terminator sequence and tac promoter sequence, which is homologous to primer catarm2F for Gibson assembly.
glcDEFarm1R	gccgatgattaattgtcaacagctcgaattcaaaaaaccgcacctgggtgcggtttttgcgcggctcactcgcaacgg	
glcDEFarm2F	gacaattaatcatcggctcgtataatgtgtcagactcaataataataataaggaggtatcgaatgaatatcctgtacga	To amplify the downstream homology region for the promoter replacement of glcDEF. Primer glcDEFarm2F contains tac promoter sequence, which is homologous to primer catarm1R for Gibson assembly. Primer glcDEFarm2R contains homologous sequence with pK18mobsacB backbone for Gibson assembly.
glcDEFarm2R	CCTGCAGGTCGACTCTAGAGGATCCctcgcagtcgtcgtaaacat	

pBBRF	TAATATCGAATTCCTGCAGCCCG	To amplify the plasmid backbone of pBBR1MCS-2 for protein expression.
pBBRR	CATAGCTGTTTCCTGTGTGA	
LCCF	CAATTTCACACAGGAAACAGCTA TGAGCAACCCGTACCAG	To amplify codon optimized LCCICCG. The primer pairs each contain homologous sequences with pBBR1MCS-2 backbone for Gibson assembly.
LCCR	CCCGGGCTGCAGGAATTCGATAT TACTGGCAGTGGCGGTTGT	

Table S2. Parameters for MA purification.

	**1**	**2**	**3**
Initial concentration of MA solution(g/L)	3.91	9.7	20.44
Initial volume of MA solution (mL)	225	150	85
Final mass of purified MA (g)	0.58	0.98	1.25
Net recovery of MA	65.43%	67.39%	71.85%
Final purity of MA	99.54 ± 0.27%	99.33 ± 0.08%	99.08 ± 0.06%

References

1. Alonso Villela, S.M.; Kraiem, H.; Bouhaouala-Zahar, B.; Bideaux, C.; Aceves Lara, C.A.; Fillaudeau, L. A protocol for recombinant protein quantification by densitometry. *Microbiologyopen* **2020**, *9*, 1175-1182, doi:10.1002/mbo3.1027.
